# Editorial: The Biological and Clinical Aspects of HLA-G

**DOI:** 10.3389/fimmu.2021.649344

**Published:** 2021-02-19

**Authors:** Joel LeMaoult, Wei-Hua Yan

**Affiliations:** ^1^CEA, DRF-Francois Jacob Institute, Research Division in Hematology and Immunology (SRHI), Saint-Louis Hospital, Paris, France; ^2^Université de Paris, IRSL, UMRS 976, Paris, France; ^3^Medical Research Center, Taizhou Hospital of Zhejiang Province, Wenzhou Medical University, Linhai, China; ^4^Key Laboratory of Minimally Invasive Techniques & Rapid Rehabilitation of Digestive System Tumor of Zhejiang Province, Taizhou Hospital of Zhejiang Province, Linhai, China

**Keywords:** HLA-G, receptor, immune cells, pregnancy, cancer, tolerance

In this Research Topic, we hosted eight in-depth reviews, mini reviews, and original research articles on the biological and clinical aspects of HLA-G. This could be unattainable without the enthusiastic involvement of all contributing authors, participating reviewers and the assistance from the staff of Frontiers in Immunology.

HLA-G belongs to the non-classical HLA class I family. HLA-G features limited genetic variation, very restricted tissue expression, and immune tolerogenic functions, being a ligand of immune inhibitory receptors. HLA-G is now recognized as an important immune checkpoint. Moreover, at least seven isoforms (membrane-bound isoforms: HLA-G1~HLA-G4; soluble isoforms: HLA-G5~HLA-G7), can be generated due to its primary transcript alternative splicing. Since *HLA-G* gene had been identified in 1987 (1) and HLA-G protein expression first observed in extravillious cytotrophoblasts in 1990 (2), both genetic and molecular characteristics, and biological functions of HLA-G have been thoroughly investigated. Even though HLA-G main site of expression it the fetal cytotrphoblast, physiological expression in adults was reported in stem cells and some progenitor cells, somatic cells within immune provoleged tissues, and some immune cells. Furthermore, HLA-G ectopic expression is induced in a variety of pathological conditions. The immune suppressive functions of HLA-G are mediated by the signaling between HLA-G and the ILT-2 and ILT-4 receptors. The importance of this interaction has been well-described in a broad range of clinical settings such as reproduction, infection, autoimmune disease and cancer. HLA-G/ILT is a promising immune checkpoint, and the first phase I clinical trial for a new anti-HLA-G antibody started in 2020 in advanced solid cancer patients (3). In the context of pregnancy, HLA-G interacts with another receptor, KIR2DL4, that is principally expressed by uterine NK cells. Interaction of HLA-G with KIR2DL4 is clearly different from that with ILT-2 and ILT-4, and its role in pregnancy is currently emerging.

In this Research Topic, different aspects of the latest advances regarding HLA-G have been reviewed and explored including the significance of *HLA-G* genetic variability in HLA-G expression and disease predisposition, the roles of HLA-G in fetal-maternal immune tolerance, the neo- and heterogeneous expression of HLA-G in cancers, and the cellular and extracellular HLA-G expression in the regulation of various immune cell functions.

The regulation of HLA-G expression is multifactorial which can be affected by HLA-G genetic variability, post-transcriptional regulation and intracellular and extracellular microenvironmental signals. The predictive, diagnostic, and prognostic significance of HLA-G genotype and/or protein expression has been investigate in a wide range of clinical settings. Amodio and Gregori elaborate how HLA-G protein expression regulated by the polymorphisms in the 5′-upstream regulatory region (5′-URR), coding region and in the 3′-untranslated region (3′-UTR) through transcriptional and posttranscriptional regulation, and discussed the most studied HLA-G polymorphism 14-bp INS/DEL in association with diseases. They also recommend future approaches for the HLA-G polymorphism/protein expression/disease association studies. Xu et al. illustrate certain HLA-G polymorphisms as risk factors for the human papillomavirus infection and HLA-G expression in cervical cancer carcinogenesis. Signaling between HLA-G and its receptor engagement is the key requirement for the immune regulatory function of HLA-G. HLA-G and its receptor signaling can exert immune suppression with detrimental effects which favors cancer and virus infected cells by allowing them to escape from immune surveillance and attack, while beneficial in promoting immune tolerance for fetal-maternal or transplants acceptance. Contini et al. review the HLA-G expressing immune cells in physiological conditions, and both in autoimmune and non-autoimmune diseases, indicating potential roles of HLA-G positive immune subsets involvement in the pathogenesis of immune mediated diseases. Wu et al. report that HLA-G, and HLA-G-expressing tolerogenic DC-10 through ILT-2 pathway inhibit both human and murine invariant natural killer T (iNKT) cell activation. Schwich et al. explore the functions of two different soluble HLA-G forms, the purified sHLA-G1 protein and extracellular vesicles with or without HLA-G molecule, on the ILT-2 positive and negative CD8+ T cells. sHLA-G1 and HLA-G_EV_ differentially induce immune-exhausted or immune-suppressive phenotye in ILT-2 positive CD8+ T cells and ILT-2 negative CD8+ T cells, respectively. This finding indicate that sHLA-G1 and HLA-G_EV_ affect ILT-2 positive and ILT-2 negative CD8+ T cells complementary. Xu et al. showcase three important roles of extravillous trophoblast expressed HLA-G on the regualtion of immune cell subsets. First, HLA-G/ILT-2 and HLA-G/KIR2DL4 signaling induces immune cell producing proangiogenic cytokines to promote the spiral artery remodeling. Second, HLA-G/ILT-2/4 and HLA-G/KIR2DL4 signaling supresses the cytotoxicity of immune cells to maintain the fetal-maternal immune tolerance. Finally, HLA-G/ILT-2 and HLA-G/KIR2DL4 signaling induces the production of growth-promoting factors to favor fetal growth. Clinical significance of HLA-G neo-expression in cancers and its relation to advanced disease stage, tumor metastasis and poor prognosis in many tumors has been well-established, and a clinical trial with a monoclonal anti-HLA-G antibody to block the HLA-G/ILTs interaction was initiated recently (3). Zhang et al. demonstrate that intratumor heterogeneity of HLA-G expression is a common phenomenon in cancers, and that the degree of HLA-G expression detection varies dramatically with different antibodies used to probe. Loustau et al. discuss the advance of HLA-G neo-expression and clinical relevance in various tumor types, and point out the limitation such as more specific anti-HLA-G antibodies are extremely necessary for future in-depth studies on neo-expression of HLA-G in cancers.

In summary, this special issue highlights the current advances regarding the biological and clinical importance of HLA-G in various physio-pathological situations. Undoubtedly, HLA-G is a promising biomarker and therapeutic target for different diseases, even though isoform-specific antibodies are still lacking, which prevents advances in characterizing their clinical significance.

## Author Contributions

W-HY wrote the first draft of the manuscript and updated the last version. JL corrected the draft.

## Conflict of Interest

The authors declare that the research was conducted in the absence of any commercial or financial relationships that could be construed as a potential conflict of interest.
